# Safety and Effectiveness of Vedolizumab for the Treatment of Pediatric Patients with Very Early Onset Inflammatory Bowel Diseases

**DOI:** 10.3390/jcm10132997

**Published:** 2021-07-05

**Authors:** Sylwia Fabiszewska, Edyta Derda, Edyta Szymanska, Marcin Osiecki, Jaroslaw Kierkus

**Affiliations:** Department of Gastroenterology, Hepatology, Feeding Disorders and Pediatrics, The Children’s Memorial Health Institute, 04-730 Warsaw, Poland; s.fabiszewska@ipczd.pl (S.F.); e.derda@ipczd.pl (E.D.); m.osiecki@ipczd.pl (M.O.); j.kierkus@ipczd.pl (J.K.)

**Keywords:** premedication, infusion reaction, infliximab, inflammatory bowel disease

## Abstract

**Background:** Vedolizumab (vedo) is effective for induction and maintenance of remission in adults with inflammatory bowel disease (IBD). Pediatric data are still limited, especially for the youngest children with very early onset disease (VEO-IBD). The aim of this study was to assess the safety and efficacy of vedo in VEO-IBD. **Methods:** We performed a retrospective review of pediatric IBD patients with VEO-IBD (defined as aged <6 years) receiving vedo. Data on demographics, disease behavior, activity, and previous treatments/surgeries were collected. Disease activity was assessed using the pediatric Crohn’s disease (CD) activity index (PCDAI) for CD or pediatric ulcerative colitis (UC) activity index (PUCAI) for UC. Primary outcome was clinical response after induction therapy with vedolizumab (4th dose week). It was defined as a decrease in PCDAI of at least 12.5 points between baseline and 4th dose week for CD, and a decrease in PUCAI of at least 20 points between baseline and this time for UC. Descriptive statistics were performed to analyze the data. **Results:** The study included 16 patients with VEO-IBD who have received vedo: 4/16 (25%) with CD, and 12/16 (75%) with UC at the median age of diagnosis 33.7 months (6.6 months–4.5 years). Median age at vedo initiation was 6.5 years (2.2–16.5 years). Among the analyzed individuals, 56.25% had failed more than one anti-tumor necrosis factor (TNF) alfa agent. Clinical response at 4th dose week was observed in 9/16 (56.3%) patients: mean baseline PCDAI score was 34.4 ± 1.9 and 10.6 ± 1.8 after induction therapy with vedo, while PUCAI score was 26 ± 6 vs. 18 ± 8, respectively. There was improvement in patients’ nutritional state: at baseline 2/16 (12.5%) children had body mass index (BMI) below 1 percentile and no child had such BMI after induction therapy with vedo. No infusion reactions or serious adverse events/infections were reported. **Conclusion:** Vedolizumab is safe and effective in the medical management of pediatric patients with VEO-IBD.

## 1. Introduction

The incidence of pediatric inflammatory bowel disease (IBD) is increasing worldwide, and the age of onset has become younger [1]. Very early onset IBD (VEO-IBD) is defined as disease diagnosis under the age of 6 years, and it represents a unique subset of IBD patients [2]. Some of VEO-IBD patients present with immunodeficiency and have genetic causes of their disease which means that loss of function genetic mutations involving immune and/or cytokines pathways lead to development of intestinal inflammation [3].

Most of these young patients present with a highly severe course of IBD and thus require more aggressive treatment due to the failure to both conventional and biological therapies [4].

Therefore, novel medical strategies are absolutely necessary, particularly for this specific group of VEO-IBD.

Monoclonal antibodies against tumor necrosis factor alpha (TNFα), such as infliximab (IFX) or adalimumab (ADA), are safe and effective in induction and maintenance of remission in moderate to severe pediatric Crohn’s disease (CD) and ulcerative colitis (UC) patients [5,6]. However, up to one-third of patients are primary non-responders to TNFα antagonist therapy and about 20% of primary responders may develop loss of response per year [7,8]. Moreover, patients who fail one TNFα antagonist are less likely to respond to a second agent, and even those with sustained response may need to discontinue therapy because of infusion reactions or other adverse effects [4].

Vedolizumab (vedo) is a humanized anti-α4β7 integrin, immunoglobulin G1 monoclonal antibody. It may be an alternative for patients with severe IBD who have failed treatment with a TNFα antagonist [9]. This biological agent downregulates intestinal inflammation by specifically inhibiting intestinal T-lymphocyte migration into the tissue. Therefore, its mechanism of action is restricted to the gastrointestinal tract, which potentially decreases the risks of systemic immunosuppression that leads to increased infection rate and malignancies observed in other IBD therapies [10].

Results from adult studies (GEMINI 1 and GEMINI 2 trials) have demonstrated the safety and effectiveness of vedo in induction and maintenance of remission in both UC and CD, with slightly better clinical outcomes in UC [11,12].

Data on vedo treatment in pediatric IBD population is still lacking and, so far, there has been no study performed on patients with VEO-IBD.

Due to the mechanism of action indicating the superior safety profile of this anti-integrin therapy, vedo seems to be a promising biological agent in pediatric IBD and is certainly worth further evaluation, especially in the group of youngest patients.

The aim of this study was to evaluate safety and efficacy of vedo in the treatment of pediatric patients with VEO-IBD.

## 2. Patients and Methods

This is a single-center retrospective observational cohort study of 16 children under the age of 6 years at diagnosis of IBD (both CD and UC) with a severe course of the disease refractory to both standard therapies and anti-TNF-alfa treatment. In this group, vedo was introduced at any age as a “rescue therapy”. Drug’s doses were either 150 mg or 300 mg depending on patient’s weight, and the infusions’ schedule was: 0, 2, and 6 weeks of induction therapy followed by a maintenance phase at 8-week intervals.

Data on demographics, disease behavior, activity, and previous treatments/surgeries were collected from patients’ medical charts. Disease activity was assessed using the pediatric Crohn’s disease activity index (PCDAI) for CD or pediatric ulcerative colitis activity index (PUCAI) for UC. Mucosal healing (MH) was assessed using fecal calprotectin (FCP) which was measured with enzyme-linked immunosorbent assay (ELISA) test kits and normal values were <50 μg/g.

The primary outcome of the study was:

- Clinical response after induction therapy with vedo (4th dose week) defined as a decrease in PCDAI of at least 12.5 points between baseline and 4th dose week for CD, and for UC a decrease in PUCAI of at least 20 points between baseline and this time.

The secondary outcomes included:

- Clinical remission after induction phase (4th dose week) and maintenance phase (10th dose week) of vedo defined as PCDAI ≤10 points for CD or PUCAI ≤10 points for UC.
- Improvement in patients’ nutritional status after induction and maintenance treatment with vedo assessed by body mass index (BMI) score.
- Improvement in laboratory parameters after induction and maintenance treatment with vedo.
- FCP was used as a surrogate marker of MH—a statistically significant decrease in FCP level between baseline and after vedo commencement was considered as MH.

To analyze the data, descriptive statistics based on intension to treat (ITT) analysis were performed.

All legal guardians provided written informed consent for the treatment with vedo.

Vedolizumab is approved in Poland for adult patients with IBD. It is used it for children as a rescue therapy and thus an agreement of the National Consultant of Pediatric Gastroenterology is obligatory.

The study meets the standards of Helsinki and the National Consultant on Pediatric Gastroenterology agreed to administer the drug.

## 3. Results

### 3.1. Patients’ Characteristics

Sixteen patients with IBD were included in the study: 4/16 (25%) with CD, and 12/16 (75%) with UC. Median age at disease diagnosis was 33.7 months (6.6 months–4.5 years). Median age at vedo initiation was 6.5 years (2.2–16.5 years). Among these individuals 15/16 (93.75%) had previously received infliximab (IFX), 9/16 (56.25%) had been treated with adalimumab (ADA), and 4/16 (25%) had had an exposure to other biologic therapies, 1/16 (6.25%) was biologic-naive. The most common reason for discontinuation of anti-TNF-alfa therapy was primary non-response: IFX—9/15 participants (60%), ADA—6/9 (66.7%). At baseline 8/16 (50%) patients received concomitant therapy with systemic steroids and 9/16 (56,3%) with immunosuppression (IMM)—either azathioprine (5 patients—AZA) or methotrexate (4 patients—MTX). After induction therapy 6/14 (42,9%) children continued steroids and 8/14 (57,1%) IMM (4 patients—MTX, 4 patients—AZA) (Table S1).

### 3.2. Outcomes

The response at the 4th dose week (induction phase) was observed in 9/16 (56.3%): 6 with UC and 3 with CD. Additionally, in UC group at 4th dose week 3 patients achieved clinical remission and 4 patients fulfill the criteria of clinical response and clinical remission. In CD group at 4th dose week 1 patient achieved clinical remission and 1 patient fulfilled the criteria of clinical response and clinical remission. Two patients did not respond to vedo therapy and therefore they did not continue the treatment. The mean PCDAI score at initiation of vedo therapy for the CD patients was 34.4 ± 1.9, while the mean PUCAI for children with UC was 26 ± 6. Overall, there was a significant decrease in both clinical indexes from baseline to each follow-up visit, at 4th and 10th dose week: PCDAI—18.1 ± 6.8, 10.6 ± 1.8, and PUCAI—18 ± 8, 17 ± 7, respectively (Figure S1). Four patients with UC had PUCAI = 0 after induction treatment with vedo.

Six patients (37.5%) either did not respond or lost response to vedo: 2 patients did not respond to induction therapy, and 4 children lost response—after the 4th, 8th, 9th and 10th doses of vedo, respectively.

All patients improved their nutritional status: 2/16 individuals (12.5%) had BMI <1 percentile at baseline and none at 4th dose week. Thirteen (81.25%) children had median baseline BMI score at 40th percentile, while all of them achieved this BMI score after induction therapy with vedo (Table S2).

Overall, there was an improvement in laboratory parameters: mean baseline hemoglobin level was 10.5 g/dL and 11.8 g/dL after 3 doses of vedo, and mean serum albumin was 38.5 g/L vs. 42.9 g/L respectively. A decrease in inflammatory markers was reported: mean ESR at week 0 was 29 mm/h vs. 18 mm/h after induction therapy (Table S2).

During our study, 8/16 (50%) patients were tested for FCP (Figure S2). Unfortunately, due to the small sample size we cannot draw any clear conclusions on the impact of vedo treatment on FCP levels.

Three patients still required surgery during vedo therapy: 2 patients underwent total colectomy and 1 had subtotal resection of the intestine (Table S3).

### 3.3. Safety

Generally, vedo therapy was safe and well-tolerated by our patients. No infusion reactions or serious adverse events (AEs)/infections were reported during the whole treatment. After induction phase only one patient (1/16–6.3%) developed infection of the upper respiratory tract, however we do not know whether it was related to vedo. After nine doses of vedo, 2 patients (12.5%) complained about arthralgia (Table S2).

## 4. Discussion

In this first study on vedo treatment in children with VEO-IBD we have demonstrated the safety and effectiveness of this anti-integrin agent in the studied group—clinical response after induction therapy with three doses of vedo was observed in more than 40% of patients. Our results are comparable to the outcomes reported so far for the older pediatric patients, which is very satisfactory regarding more severe course of VEO-IBD and thus worse response to treatment.

In the retrospective multi-center study of the pediatric IBD Porto Group of European Society of Pediatric Gastroenterology, Hepatology and Nutrition (ESPGHAN) demonstrating experience with vedo in pediatric population, the remission rate at week 14 was 37% in UC, and 14% in CD, while by last follow-up it was 39% in UC and 24% in CD respectively. However, children in this group were much older than our patients—their mean age at vedo commencement was 14.5 ± 2.8 years, while it was 7.24 years in our cohort. Alike our patients, all of those children were previously treated with anti-TNF-alfa agent (28% primary failure, 53% secondary failure) [13]. However, 17% children still required surgery, including a colectomy for UC. An interesting observation was that concomitant immunomodulatory therapy did not affect remission rate [42% vs. 35%; *p* = 0.35 at Week 22]. In this study, only 3 of 16 children who underwent endoscopic evaluation had MH after treatment (19%). What is most important, only three minor drug-related AEs were observed [10].

In our study 3 patient required surgery and 2 of them underwent toral colectomy. Concomitant treatment with either systemic steroids or IMM was continued in 3/9 (33.33%) children at 4th dose visit, and 6/9 (66.7%) of participants at the 10th dose week. This observation is consistent with the PORTO group results. Due to the very young age of study participants, we decided to assess MH using a non-invasive biomarker, FCP, not an endoscopy which requires general anesthesia and is more stressful for children. The reason for this was that FCP has been proven to correlate best with endoscopic improvement (better than clinical indexes) and, therefore, is considered to be the marker of MH and has been used as such in [14]. Alike in the PORTO group study, significant improvement of the intestinal permeability was not achieved during vedo treatment in our patients, which demonstrates that deeper remission requires more time than clinical and laboratory improvements. Moreover, data published so far show that full effect of vedo is observed between 6 and even 14 weeks of treatment, which indicates that MH may be achieved later than with other biologics.

Nonetheless, it is important to underline that VEO-IBD is characterized by a more severe course than typical pediatric disease and, therefore, the outcomes of medical management may be worse [15]. Similarly in the Porto Group study, vedo was safe and well-tolerated by our patients—during the whole therapy only one child (1/16–6.3%) developed infection of the upper respiratory tract, and 2 patients (12.5%) complained about arthralgia. However, we do not know whether these AEs were related to vedo.

Another multi-center study published in 2016 showing experience with vedo in pediatric IBD has proven its effectiveness and safety in this population [16]. In this trial which included 58% CD and 42% UC patients with median age at vedo initiation of 14.9 (range 7–17) years, week 14 remission rates for UC and CD were 76% and 42%, respectively. In this cohort 90% of patients had failed more than 1 anti-TNF agent. Among anti–TNF-naive patients 80% of them experienced week 14 remission. At week 22, anti–TNF-naive patients had higher remission rates than TNF-exposed patients (100% versus 45%, *p* = 0.04). There were no infusion reactions or serious AEs [12].

These outcomes confirm two major observations from adults’ trials—vedo is more effective in UC and anti-TNF-alfa naïve patients have better response to this anti-integrin agent, which is also consistent with results of our study. This may be partially explained by the fact that anti-TNF agents downregulate MadCAM-1 expression which may be the reason that CD patients with previous anti-TNF exposure in the GEMINI studies required a longer duration of vedo treatment to achieve remission [8,9,17].

In the study by Conrad et al. evaluating outcomes of vedo therapy in severe pediatric IBD including 21 subjects, 16 with CD, clinical response was observed in 6/19 (31.6%) of the patients at week 6 and in 11/19 (57.9%) by week 22. There were no infusion reactions. Vedolizumab was discontinued in 2 patients because of severe colitis, requiring surgical intervention [18]. These response rates are comparable to our results.

Standard dosing of vedo in adult patients is 300 mg per infusion, and no specific guidelines exist for pediatric dosing. Children, smaller in size and weight, may require an individualized dose. Our patients received either 150 mg or 300 mg doses of vedo depending on their body mass—due to the younger age of participants in our study than in other pediatric trials, and thus lower body mass, we had to adjust dosing and lower it in the youngest children.

Recent pharmacokinetic data have demonstrated significant correlation between higher vedo drug levels and clinical response in IBD patients which may suggest that, alike with IFX, shortening infusion interval from 8 to 4 weeks should lead to an improved effect [19,20,21].

In our study intervals between vedo infusions were standard—all patients have received the drug every 8 weeks during the maintenance phase.

A very important issue is the drug’s safety. Although GEMINI 1 revealed no difference in AE rates between vedo and placebo, [8] GEMINI 2 demonstrated a higher incidence of nasopharyngitis with vedo than with placebo [12.3% vs. 8%]. [9] The adult US VICTORY study of 212 patients reported enteric infections (5 per 100 patient-year follow-up [PYF]), sinopulmonary infections [4.4 per 100 PYF] and arthralgia [3.1 per 100 PYF], among other less common AEs [22]. Other cohorts report infections from 0% to 25%, nasopharyngitis 0–23%, arthralgia 2–20%, and one report of anaphylaxis and rash [23,24,25].

In our study no AEs or serious infections were reported, only minor medical events have been observed and their rate was low—3/16 (18.8%) patients had either respiratory tract infection (1/3) or arthralgia (2/3) during the whole therapy. Therefore, vedo seems to be very safe in children, even the youngest ones.

The limitation of our study is its retrospective design and relatively small cohort. Also, not all patients before and after vedo commencement had their FCP assessed which made it difficult to draw proper conclusions. However, it is still the only study that includes not only pediatric and early onset but also VEO-IBD patients.

To summarize, VEO-IBD group that was analyzed in our study is a unique subset of pediatric IBD patients. It is characterized by more severe course and aggressive behavior with a high treatment failure rate to both conventional and biologic therapies. In the majority of our patients, vedo was applied as a rescue therapy. Therefore, response and remission rates may be lower than in older/adult patients.

## 5. Conclusions

Vedolizumab is safe and effective for the treatment of VEO-IBD. This anti-integrin therapy provides improvement in patients’ nutritional state and in their laboratory parameters. However, achievement of MH may require more time than with other biologics due to the drug’s delayed full effect.

## Data Availability

Not applicable.

## Supplementary Materials

The following are available online at https://www.mdpi.com/article/10.3390/jcm10132997/s1 Table S1. Baseline characteristics of the study group. Table S2. Patient’s characteristics after vedolizumab initiation compering to baseline. Table S3. Relationship between vedolizumab and surgery in 3 patients who underwent surgery. Figure S1. Patients’ disease clinical activity (PCDAI for CD and PUCAI for UC) between baseline and after vedolizumab commencement (4th and 10th week dose). Figure S2. Values of fecal calprotectin for 8 patients tested for this marker before and after vedolizumab commencement.
