# Supplementary material for: Safety and Effectiveness of Vedolizumab for the Treatment of Pediatric Patients with Very Early Onset Inflammatory Bowel Diseases
Source: J Clin Med. 2021 Jul 5;10(13):2997. doi: 10.3390/jcm10132997 (PMC8268556; doi:10.3390/jcm10132997)
Supplement: Supplementary file 1 [file jcm-10-02997-s001.zip › jcm-1219452-supplementary.pdf]

Table S1. Baseline characteristics of the study group.

Baseline	Total cohort (N=16)
Males	10 (62,5%)
Female	6 (37.5%)
Median age at disease diagnosis; mth, yrs	33.7 mth (6.6 mth – 4.5 yr)
Median age range at vedo initiation; yrs	6.5 (2.2 – 16.5)
Crohn’s disease	4/16 (25%)
Ulcerative colitis	12/16 (75%)
IFX exposure	15/16 (93.75%)
ADA exposure	9/16 (56.3%)
Other biologics exposure	4/16 (25%)
**IFX discontinuation reason:**<br>- non-response<br>- allergic reaction<br>- loss of response<br>- other	9/15 (60%)<br>3/15 (20%)<br>1/15 (6,7%)<br>2/15 (13,3%)
**ADA discontinuation reason:**	

- non-response<br>- allergic reaction<br>- loss of response<br>- other	6/9 (66.7%)<br>0/9<br>2/9 (22.2%)<br>1/9 (11.1%)
**Previous conventional therapies**<br>- Systemic steroids<br>- Budesonide MMX<br>- 5-ASA<br>- Azathioprine therapy<br>- Cyclosporin therapy	 16 (100%)<br>8 (50%)<br>15 (93.8%)<br>14 (87.5%)<br>10 (62.5%)
FCP; ug/g	1943.1

Abbreviations:

Months – mth

Years – yr

Vedolizumab - vedo

Infliximab – IFX

Adalimumab – ADA

5-aminosalicylate acid – 5-ASA

Body Mass Index – BMI

Pediatric Crohn's disease Activity Index - PCDAI

Pediatric Ulcerative Colitis Index – PUCAI

Fecal calprotectin – FCP

Table S2. Patient's characteristics after vedolizumab initiation compering to baseline.

N=16	Baseline	4th dose week	10th dose week
Median BMI: percentile	40	40	39
**Laboratory parameters**			
- mean Hemoglobin; mmol/l	10.5	11.8	11,7
- mean ESR; mm/h	29	18	19
- mean Albumin; g/l	38.5	42.9	41,3
**Adverse events/ No of patients**<br>-infection of the upper respiratory tract	0	1	-
- arthralgia	0	0	2

PCDAI - pediatric Crohn's disease activity index.

PUCAI - Pediatric Ulcerative Colitis Index

BMI – body mass index

ESR - Erythrocyte sedimentation rate

Table S3. Relationship between vedolizumab and surgery in 3 patients who underwent surgery.

Patient	Number of doses of vedolizumab	Number of weeks between vedolizumab commencement and surgery procedure	Number of weeks between last dose of vedolizumab and surgery procedure
Patient A	3	13	6
Patient B	3	108	102
Patient C	9	58	6

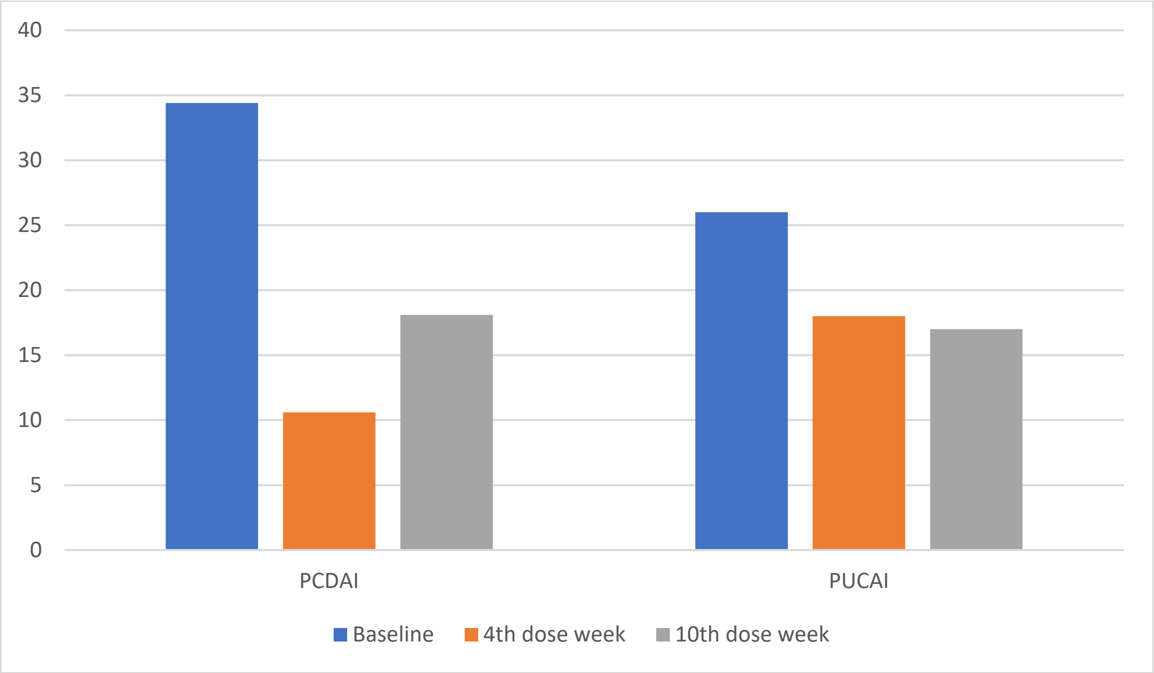

Figure S1. Patients' disease clinical activity (PCDAI for CD and PUCAI for UC) between baseline and after vedolizumab commencement (4th and 10th week dose).

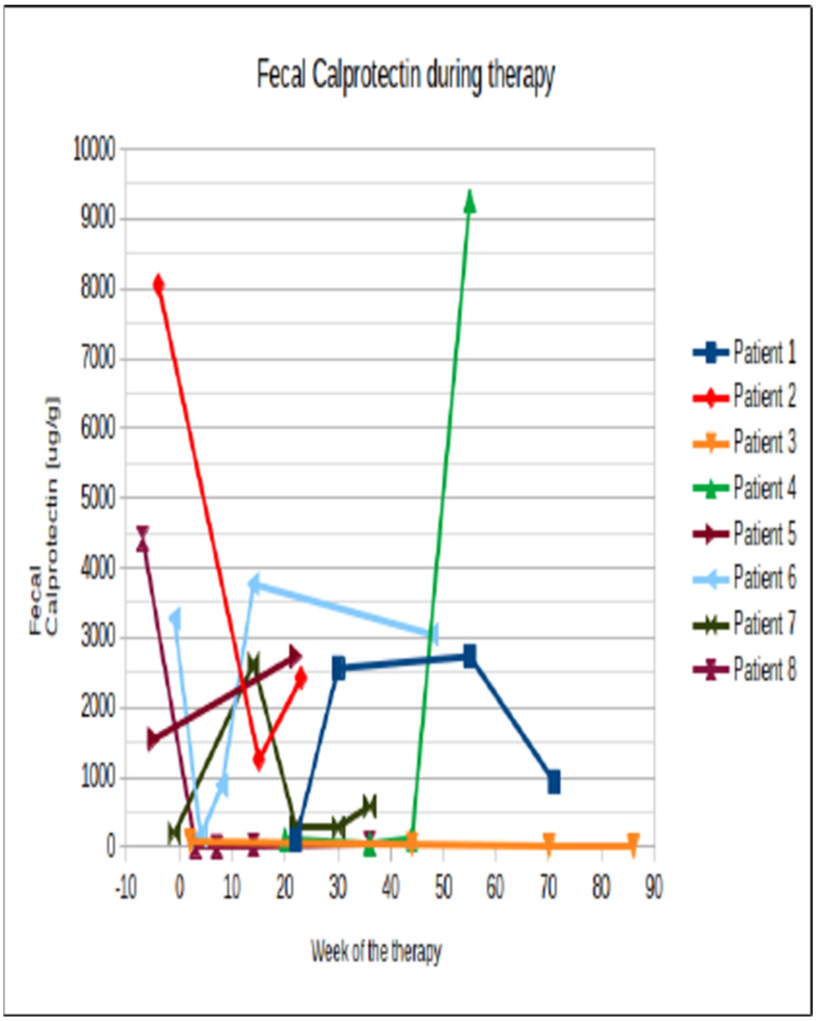

Figure S2. Values of fecal calprotectin for 8 patients tested for this marker before and after vedolizumab commencement.
